# Exploring the power of spectrophotometric technique in determination of oxytetracycline and lidocaine in their pharmaceutical dosage form as well as in the presence of toxic lidocaine impurity: univariate versus multivariate analysis

**DOI:** 10.1186/s13065-024-01373-2

**Published:** 2025-01-16

**Authors:** Naglaa Ahmed, Ahmed Hemdan, Hala ZaaZaa, Maha Galal

**Affiliations:** 1 Pharmaceutical Analytical Chemistry Department, Faculty of Pharmacy, Ahram Canadian University, Giza, Egypt; 2 Department of Pharmaceutical Chemistry, Faculty of Pharmacy, Kuwait University, Kuwait City, Kuwait; 3 Pharmaceutical Analytical Chemistry Department, Faculty of Pharmacy, Cairo University, Cairo, Egypt

**Keywords:** Lidocaine, Oxytetracycline, 2,6-dimethylaniline, Univariate and multivariate methods, Application to pharmaceutical dosage form, Statistical comparison

## Abstract

Lidocaine poses challenges when it comes to direct spectrophotometric measurement due to the lack of sharp peak within its spectra in zero-order. This lack of a distinct peak makes it difficult to accurately quantify lidocaine using traditional direct spectrophotometric methods. In our study, different univariate and multivariate spectrophotometric techniques have been established and their validity has been assessed for the determination of the mixture of Lidocaine HCl (LD), Oxytetracycline HCl (OTC) together with LD carcinogenic impurity [2,6- dimethylaniline] DMA. LD was resolved from the other two components using ratio difference and derivative ratio methods. OTC was determined in zero- order at 360 nm and by using constant value and concentration value methods, while DMA was determined by using constant multiplication at 237 nm as well as by using constant value and concentration value methods after elimination of OTC by ratio subtraction technique. Moreover, Partial Least Squares and Principal Component Regression multivariate approaches were applied to quantify and evaluate the mixture. The developed methods underwent validation following International Council for Harmonization guidelines. The validation process demonstrated that all suggested methods are accurate and selective in their measurements. Additionally, statistical analysis was conducted to compare the developed and reported methods. Furthermore, one-way analysis of variance was performed to compare both proposed and reported spectrophotometric methods.

## Introduction

**Fig. 1:**
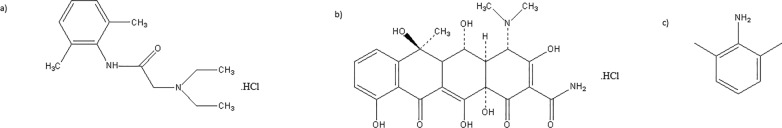
Chemical structures of **a** Lidocaine hydrochloride, **b** Oxytetracycline hydrochloride, and **c** 2,6-dimethylaniline

Oxytetracycline HCl (OTC), shown in (Fig. 1), is known for its broad antibacterial activity. It can effectively target and inhibit the growth of a diverse range of bacteria, encompassing Gram-negative and Gram-positive types. This versatility makes it valuable in combating bacterial infections across different veterinary applications. It acts by inhibiting bacterial protein synthesis by attaching the bacterial ribosomes and inhibiting the binding of amino acids to the growing peptide chain, thereby preventing bacterial growth and reproduction [1, 2]. Lidocaine HCl (LD), shown in (Fig. 1), is an amide-type local anesthetic which is commonly utilized for anesthesia and blockage of regional nerves. It exhibits a fast onset of action, providing anesthesia within a matter of minutes. The interaction between LD and voltage-gated sodium (Na^+^) channels found in the membrane of nerve cells is considered the mechanism of action of LD. This interaction leads to the inhibition of the transient increase in Na^+^ permeability of excitable membranes. As a result, the initiation and transmission of nerve impulses are effectively impeded [3]. 2,6- dimethylaniline (DMA) is a degradation product as well as an official impurity of LD. It is also designated as LD toxic metabolite, with potential links to urinary bladder cancer and reported cases of nasal carcinogenesis in rats [4]. Therefore, our aim in the suggested methods is to separate OTC and LD from each other and from toxic LD impurity as well as to ensure that this toxic impurity does not exist in the pharmaceutical dosage form.

Chemometric is a scientific discipline that relates principles of chemistry, statistics and mathematics for extracting useful information from chemical data. It provides tools and techniques for data preprocessing, exploratory data analysis, calibration, prediction, experimental design, method validation, and process control [5–7].

Several analytical methods have been recorded for the simultaneous analysis of LD and DMA among these methods are spectrophotometry [8], potentiometry [9], voltammetry [10], high-performance liquid chromatography [11, 12] and solid phase microextraction (SPME) in conjunction with gas chromatography/mass spectrometry (GC/MS) [13]. As well as numerous methods have been recorded for determination of LD and OTC which include: spectrophotometry [14] and HPLC [15]. There is only one HPLC method in conjunction with TLC method was reported for determination of LD and OTC in the presence of DMA [16]. To the best of our knowledge, the three mentioned compounds were not simultaneously determined by any spectrophotometric method. The big challenge is that LD lacks its sharp peak in the spectrum of zero-order. The reported GC/MS and HPLC methods have disadvantages of high cost and complex instrumentation. So, our aim was the determination of LD and each component of the ternary mixture by accurate and precise spectrophotometry without the interference of two other components and without dependence on its shoulder peak. The main object of the proposed method is to compare and validate the ternary mixture assay results by utilizing direct and more sensitive univariate spectrophotometric and multivariate chemometric methods than the reported one. The methods revealed that the marketed dosage form has no contamination with the carcinogenic LD impurity DMA. Our study was validated for linearity, precision, accuracy, detection limit, and quantitation limit.

### Theoretical background

#### Constant multiplication (CM)

The use of this method is to separate binary or ternary mixtures with overlapping spectra. For example, if we have an A and B mixture, where B is more extended than A, so zero- order spectrum of B (more extended) is obtained and completely resolved from A by dividing the mixture by specific divisor of B (B’) so a new spectrum will be obtained which includes a constant region B/B’ that appears at the extended part, where there is zero contribution from component A.

To get B, the constant is multiplied by divisor spectrum (B’) as follows.

B’ *B/B’ = B [17–19].

#### Ratio difference

This method can be particularly useful in cases where the components of interest exhibit severe overlapping spectra or have similar spectral features. The method relies on that if we have an X and Y mixture and we need to determine component X, we will divide the mixture by a specific divisor derived from component Y; (Y’). This division results in a new spectrum where the peak amplitudes can be compared at two different wavelengths. To obtain the component X in the mixture the difference in peak amplitudes at these two wavelengths is then used [20].

#### Derivative ratio spectrum method

The derivative ratio approach is very useful for separating closely spaced peaks or for assessing samples with overlapping absorbance bands. It helps to resolve overlapping peaks and provides a clearer differentiation between the components present in the sample, so to resolve overlapping peaks, we divide the spectrum of a mixture of X and Y by a specific divisor of Y (Y’), and then apply the first derivative to the resulting spectrum. The division by the divisor serves to normalize the spectral contributions of Y. Taking the first derivative of the obtained spectrum further enhances the ability to resolve overlapping peaks and distinguish between the individual component [21].

#### Constant value (CV)

For mixtures that partially overlap, the constant value method is an intelligent strategy to be utilized. Finding the areas where a component does not contribute to the spectra of other components is the key to this method.

For instance, if we have mixture of A, B and C, C is more extended than B and B is more extended than A; to apply the constant value method, the mixture spectrum is divided by the spectrum of normalized divisor of the extended compound (B or C) at a specific concentration (B’or C’). This division yields a constant value at the plateau region at which there is no interference from the other components. The constant value obtained and the concentration of the extended compound (B or C) are directly proportional to each other [22].

#### Concentration value (CNV)

In spectrophotometry, CNV is considered a highly advanced approach that aims to simplify the quantification process by directly extracting the concentration of analytes from the spectral graph without the need for constructing calibration curves or regression equations. The CNV approach relies on the spectral data graphical representation to determine the analyte concentration actual value. This could involve analyzing specific features, patterns, or relationships observed in the spectral graph to infer the concentration directly.

By minimizing the manipulation steps and bypassing the calibration curve construction, the CNV approach potentially offers a more streamlined and efficient method for obtaining analyte concentrations [23–26].

## Experimental

### Instrumentation and software

Spectrophotometric measurements were performed using a double-beam UV–Vis spectrophotometer (Model J-760, Jasco, Japan) equipped with 1 cm path length matching quartz cells. For statistical computations, MATLAB for Windows 7 Math work, Inc. 2009 and PLS toolbox 2.0 Eigenvector Research Inc. 2005, developed by B. M. Wise and N. B. Gallagher for MATLAB use, were utilized for PCR and PLS determination.

### Materials and reagents

OTC HCl and LD HCl reference standards were provided by Pharma Swede Pharmaceutical Company (Egypt) while LD impurity (DMA) was purchased from Sigma Aldrich (Germany) and their purities were certified to be 99.89, 99.87 and 99.90% for LD, OTC and DMA, respectively. Acetonitrile (HPLC grade) was supplied from Sigma Aldrich. Spectropan 5® Veterinary Vial, produced by (Pharma Swede Pharmaceutical Company) and purchased from the local Egyptian market, is labeled to contain 1 mg of LD HCl and 54 mg of OTC HCl per 1 mL.

### Solutions

#### Standard stock and working solutions of OTC, LD and DMA preparation

Standard stock solutions (0.2 mg/mL) of standard OTC, LD and DMA were prepared separately using volumetric flasks 100 mL by dissolving accurately weighed 20.0 mg of each authentic drug in acetonitrile and complete with the same solvent to the mark. To prepare (100.0 μg/mL) of working standard solutions for each drug, the primary stock solutions were mixed with acetonitrile and diluted. Subsequently, various portions of the (100.0 μg/mL) working solutions of OTC, LD, and DMA were transferred into multiple 10 mL volumetric flasks, then adding acetonitrile to each flask to reach the desired volume, resulting in mixtures with varying concentrations of each drug.

### Spectral characteristics and wavelength selection

The scanning of Zero-order absorption spectrum of OTC, LD, and DMA against acetonitrile as a blank (200–400 nm) was done to determine the optimal resolution techniques for the mixture. The obtained spectra of each drug were then overlaid using the spectra manager software.

### Procedures

#### Linearity and construction of calibration curves

Three different sets of 10-mL volumetric flasks were filled with precisely measured portions of LD, OTC, and DMA that had been transferred from their appropriate working standard solutions. The flasks volume was then adjusted to the desired level using acetonitrile. Preparation of Calibration standards was done to cover concentration ranges of (1.0–9.0 µg/mL), (4.0–34.0 µg/mL) and (1.3–12.0 µg/mL) for LD, OTC, and DMA respectively. Then scanning of the samples was done within the wavelength range of 200–400 nm, and the resulting spectra (Zero-order spectra) for each component were saved.

#### Method of Constant multiplication for DMA determination

Calibration curve was constructed based on correlating the zero-order scanned spectra of DMA at λ_max_ 237 nm to the corresponding concentration and then calculating the regression equation.

#### Ratio difference method for determination of LD

Zero-order absorption spectrum was stored then divided by normalized spectrum of DMA. The acquired spectrums amplitude was then measured at 218 and 230 nm. The calibration curve was produced by calculating and plotting the difference between these two amplitudes against concentration. And finally, regression equation was calculated.

#### Derivative ratio spectrum method for determination of LD

Zero-order absorption spectrum of LD was stored then divided by normalized spectrum of DMA, after that the First derivative of the obtained spectrum was computed, and amplitudes of resulting spectra have been measured at 228 nm (with scaling factor 10 and Δ λ 4) to determine LD. Subsequently, construction of a calibration curve was done using measured amplitudes. The regression equation was then calculated.

#### Constant value for determination of OTC and DMA

The zero-order absorption spectra were stored for each drug and then divided by its normalized spectrum. A new spectrum was obtained which contained constant region. The construction of calibration curve was achieved by relating this constant to the corresponding concentrations of OTC and DMA.

### Validation

Accuracy, specificity, precision, detection limit, and quantitation limit were all validated in accordance with the International Council for Harmonization (ICH) guidelines [27].

#### Accuracy

In order to assess an accuracy of developed methods, three replicates of OTC, LD and DMA at varying concentrations were employed. Concentrations were determined using a corresponding regression equation, and the resulting % recoveries were calculated.

#### Precision

##### Repeatability and intermediate precision

To assess precision or repeatability of proposed methods, three various concentrations of OTC, LD and DMA were analyzed within the same day. Each concentration was analyzed three times by using the relevant methods. The percentage of relative standard deviations (RSDs) was then calculated to evaluate the precision of measurements. To calculate the proposed methods intermediate precision, the repetition of same procedures was done on three various days for the analysis of the three concentrations of OTC, LD and DMA. The RSDs were calculated based on the measurements obtained on different days.

#### Selectivity

Selectivity was assessed through preparation of different mixtures of the three mentioned drugs by transferring various portions of the previously created working solutions (100.0 μg/mL) of OTC, LD, and DMA into multiple 10 mL volumetric flasks. Acetonitrile was then added to each flask to reach the desired volume, resulting in mixtures with varying concentration of each drug.

#### Limit of quantitation (LOQ) and limit of detection (LOD)

Under recommendations provided by the International Council for Harmonization (ICH), there are various approaches to determine the quantitation and detection limits. In this study, we used the slope approach and the standard deviation of the intercept, to calculate LOD and LOQ, as follows:

LOD = 3.3 × SD of residuals.

LOQ = 10 × SD of residuals.

These calculations were performed to determine the minimum concentration of OTC, LD and DMA that can be reliably detected (LOD) and quantified (LOQ) using the proposed methods.

### Application to pharmaceutical dosage form

Determination of LD and OTC in their dosage form (Spectropan 5®) involved transferring 0.5 mL of the dosage form into a 100 mL volumetric flask, mixing it with acetonitrile, then complete to the mark with same solvent in order to give a concentration of (270.0 µg/mL OTC) and (5.0 µg/mL LD). After that 10 mL of the prepared solution was placed into a 100 mL volumetric flask and standard addition technique is used for LD where 2 mL of working standard solution of LD was added and volume then completed with acetonitrile to achieve concentration ratio of 27.0: 2.5 µg/mL of OTC and LD, respectively. The standard addition of LD was performed to enhance LD concentration to be within its linearity range.

### Experimental design for chemometric methods

A calibration (training) set was created to build the optimum PLS and PCR models. This set consisted of 25 synthetic mixtures, each containing varying concentration ratios of DMA, LD, and OTC ranging from (1.3–12.0 μg/mL), (1.0–9.0 μg/mL) and (4.0–34.0 μg/mL) respectively. In 10 mL volumetric flasks, the mixtures were prepared through mixing various volumes of their corresponding working standard solutions and diluting with acetonitrile to achieve the required volume. The prepared mixtures absorption spectra were then scanned in a wavelength range of 200–400 nm, using acetonitrile as the reference blank. Subsequently, the PLS and PCR models were developed based on the obtained data. The spectra data points within the range of 204–370 nm were exported to MATLAB to be analyzed, including the development of multivariate calibration models. Prior to calibration, the data of all spectra were mean-centered. For model construction, we utilize the concentration matrices and the absorbance of the training set in conjunction with the PLS Toolbox 2.0 software for the necessary calculations. A training set is a subset of data used to build a model. It is the relationship between input variables (predictions) and the output variable (Response). It is used to fine-tune the model parameters and enable it to make accurate predictions based on the input data. In PCR, the training set is used to calculate principal components through Principal Component Analysis (PCA), which focuses solely on the structure of the predictor variables, not the response variable. This is followed by performing regression on these components while, in PLS, the training set is used to extract latent variables that maximize the covariance between the predictors and the responses. A cross-validation strategy was used to find the ideal number of factors needed to construct the PLS and PCR models. This required one sample to be removed at a time and performing PCR and PLS calibrations on the 25 samples (shown in Table 1). This calibration was then used to expect the concentration of the omitted sample, and predicted and known concentration values were compared. The root mean square error of calibration (RMSEC) and root mean standard error of validation (RMSECV) were determined for every iteration, following same procedure. This process was repeated until maximum number of factors [were chosen to be 13] was reached for calculation of the optimum RMSEC and RMSECV.

**Table 1:** A five level, three-factorial experimental design showing mixtures of different concentrations of OTC, LD, and DMA in calibration and validation sets, expressed in μg/mL

Mixture no	Concentration μg/mL		
	OTC	LD	DMA
1	15	5	6
2	15	1	1.3
3	4	1	12
4^*^	4	9	4
5^*^	34	3	12
6^*^	10	9	6
7	34	5	4
8	15	3	4
9	10	3	9
10^*^	10	7	12
11	20	9	9
12	34	7	6
13	20	5	12
14	15	9	12
15	34	9	1.3
16	34	1	9
17^*^	4	7	1.3
18	20	1	6
19^*^	4	5	9
20	15	7	9
21^*^	20	7	4
22	20	3	1.3
23	10	1	4
24	4	3	6
25	10	5	1.3

^*^Validation set

## Results and discussion

DMA poses a highly toxic properties and biorefractory characteristics, The International Agency for Research on Cancer (IARC) has classified this chemical as a group 2B carcinogen [28].Spectropan-5® is a Veterinary dosage form that contains OTC and LD. This medication can be used to treat bacterial infections caused by both Gram-positive and Gram-negative bacteria. These infections commonly affect the respiratory, urinary, and gastrointestinal tracts, such as bronchopneumonia, pleuropneumonia, septicemia and actinobacillosis. Furthermore, it demonstrates efficacy against mycoplasma, and large viruses [29]. In the existing literature, no Spectrophotometric method was available for simultaneous determination of OTC, DMA, and LD. Therefore, our objective was to create reliable and precise methods for concurrently analyzing the mixture of three components. Additionally, we aimed to compare outcomes of univariate as well as multivariate analysis. Application to pharmaceutical dosage form indicates that there is no contamination with carcinogenic impurity of LD (DMA).

**Fig. 2:**
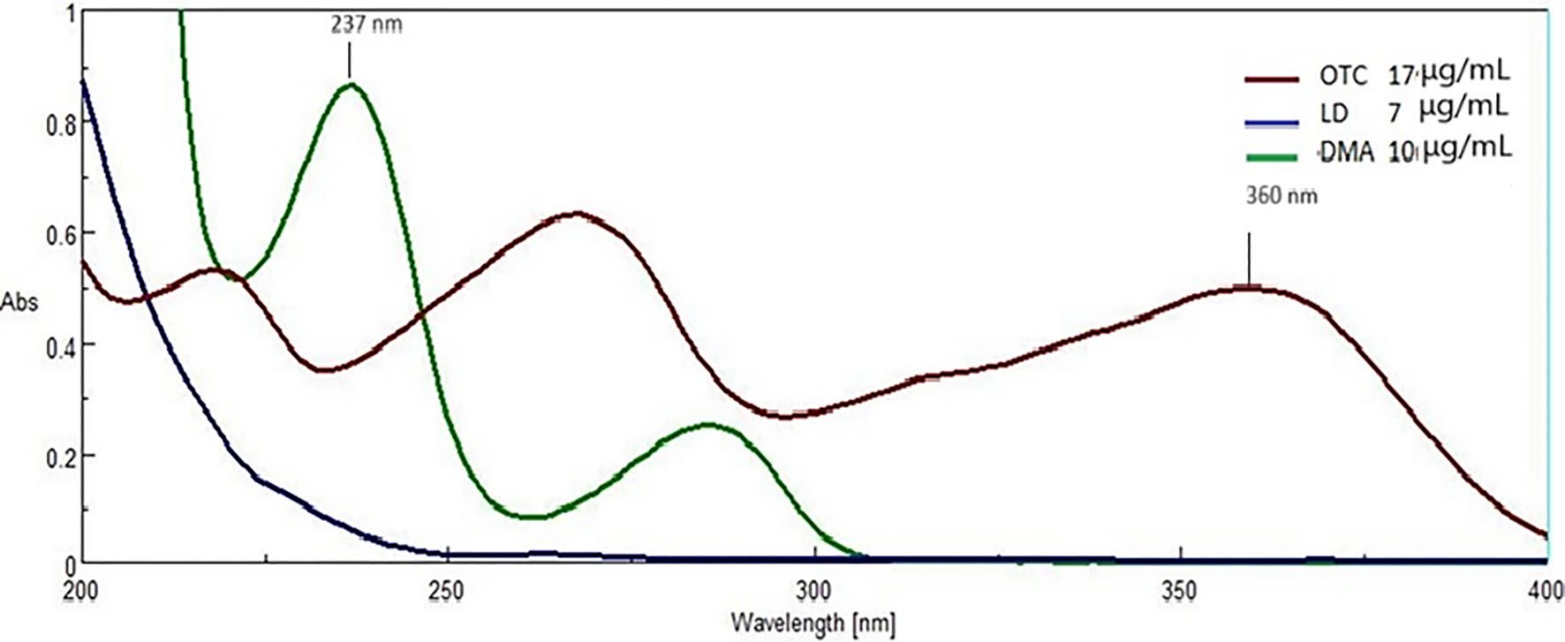
**A** Zero-order absorption UV spectra of 17.0 µg/mL Oxytetracycline, 7.0 µg/mL Lidocaine and 10.0 µg/mL 2,6- dimethylaniline in acetonitrile

As seen in Fig. 2, it is clear that the three drugs spectrum is strongly overlapped. Only OTC can be detected directly from the zero-order spectrum at 360 nm. while DMA and LD need a sensitive method to be determined as they are severely overlapped at their spectra.

## Methods development

### CM for DMA determination

After the elimination of OTC by ratio subtraction resolution technique, DMA was determined at zero-order after the removal of the interfering spectrum of LD by constant multiplication. This was done by dividing mixture spectra after OTC elimination by specific divisor Y’ (Normalized spectrum) of DMA which is the more extended one. After division, the constant present in the region where DMA is more extended is multiplied by the divisor Y’. So, DMA zero-order spectrum was obtained, making it possible to determine the concentration of DMA at 237 nm.

The following mathematical formulas could be used to summarize this:

(LD + DMA)/DMA’ = LD/DMA’ + DMA/DMA’

DMA/DMA’= Constant

So, DMA’ X Constant (DMA/DMA’) = DMA zero-order spectrum

Where DMA’ is a divisor of DMA (1 µg/mL).

### Concentration value and constant value for determination of DMA and OTC

**Fig. 3:**
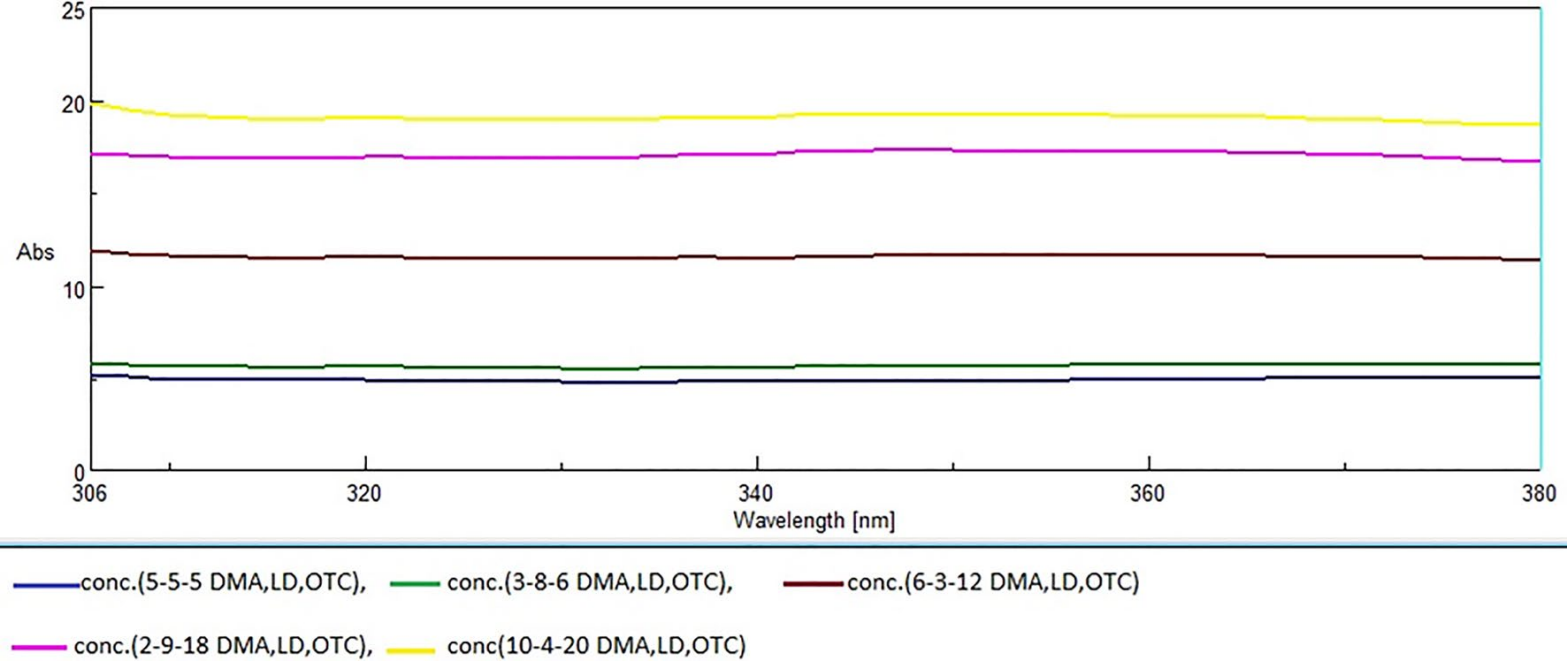
The constants resulted from the division of absorption spectra of zero- order of laboratory-prepared mixtures of Oxytetracycline, Lidocaine and 2,6- dimethylaniline by 1.0 µg /mL of Oxytetracycline as a divisor

As mentioned before, these two methods were used when we have a mixture of 3 drugs A, B and C. B is more extended than A and C is more extended than B, so OTC was determined by CV method via dividing the mixture spectrum by specific divisor of OTC (C’), after division constant region was obtained at which OTC has no contribution from either LD or DMA at region (306-380nm) (as shown in Fig. 3). In both CV and CNV methods, we employ two intelligent approaches to utilize a specific constant. Firstly, In CV we establish a calibration curve correlating the Peak Amplitude at the constant Region with the corresponding OTC concentrations. This allows us to determine the concentration of OTC by applying the regression equation derived from the curve. Secondly, in CNV, we directly obtain the concentration from the graphical representation of spectral data using the constant, without relying on the regression equation. In this case, the constant represents the concentration of OTC alone[since OTC' serves as a normalized divisor (1µg/mL)] and the extended portion of the curve where no contributions from other components exist. After the elimination of OTC by ratio subtraction, we can apply CV and CNV methods for resolution of DMA using DMA’ as normalized divisor (1µg/mL). A new spectrum was obtained at which DMA has no interference from LD at region (280–300 nm).

### Ratio difference method for determination of LD

**Fig. 4:**
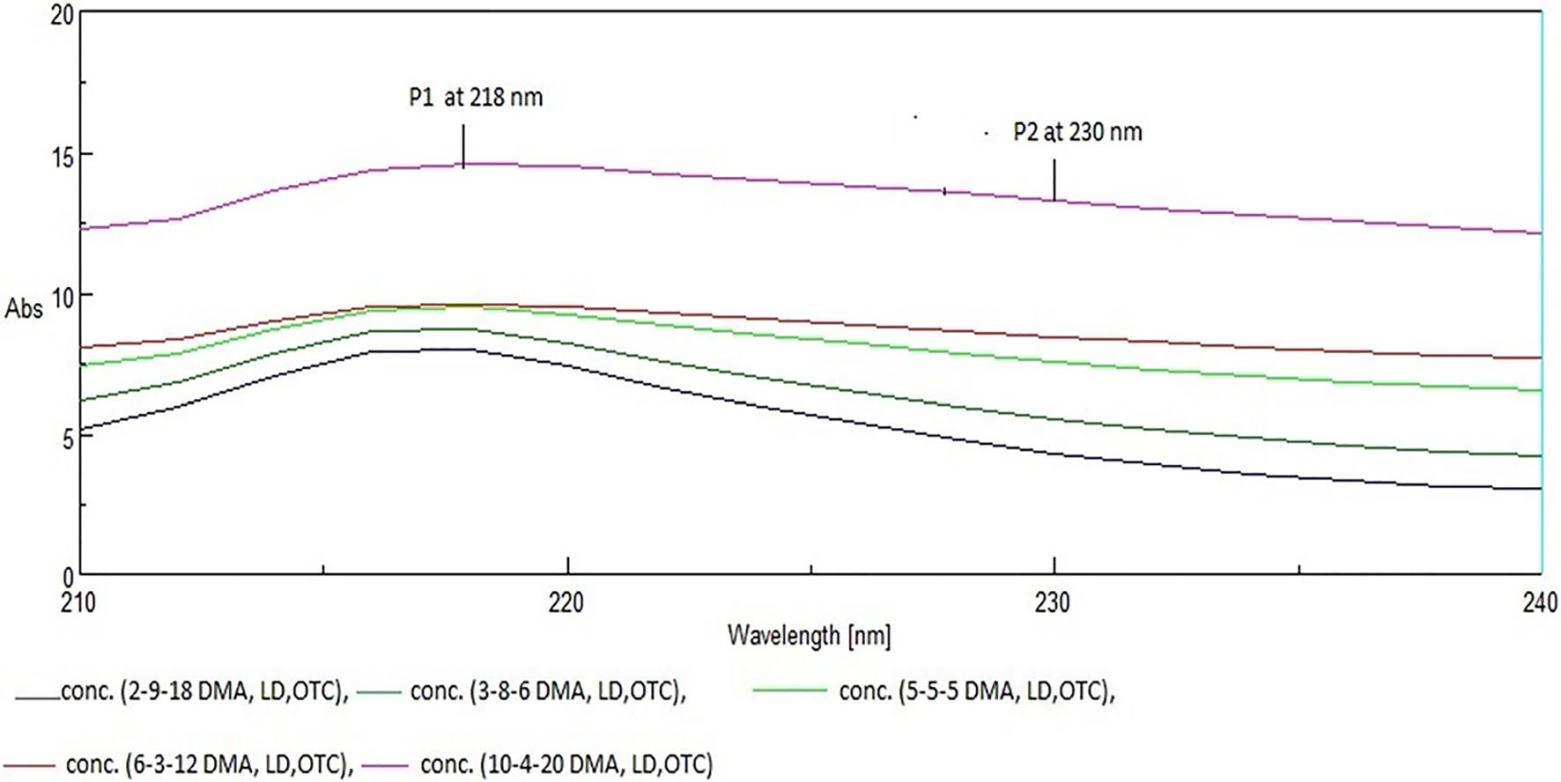
The division of zero-Dorder spectra of laboratory-prepared mixtures (after removal of oxytetracycline) by 1.0 µg /mL of 2,6- dimethylaniline as a divisor (ratio difference method)

After the elimination of OTC by ratio subtraction method, LD was resolved from DMA via ratio difference method. This involves the division of the mixture, after elimination of OTC, by normalized divisor of DMA (DMA’), then measuring the amplitude of the obtained spectrum at 218 and 230 nm (maximum and minimum amplitudes shown in Fig. 4) after that the difference between those two amplitudes was determined and recovery percent was calculated using the previously computed regression equation.

### Derivative ratio spectrum method of LD

**Fig. 5:**
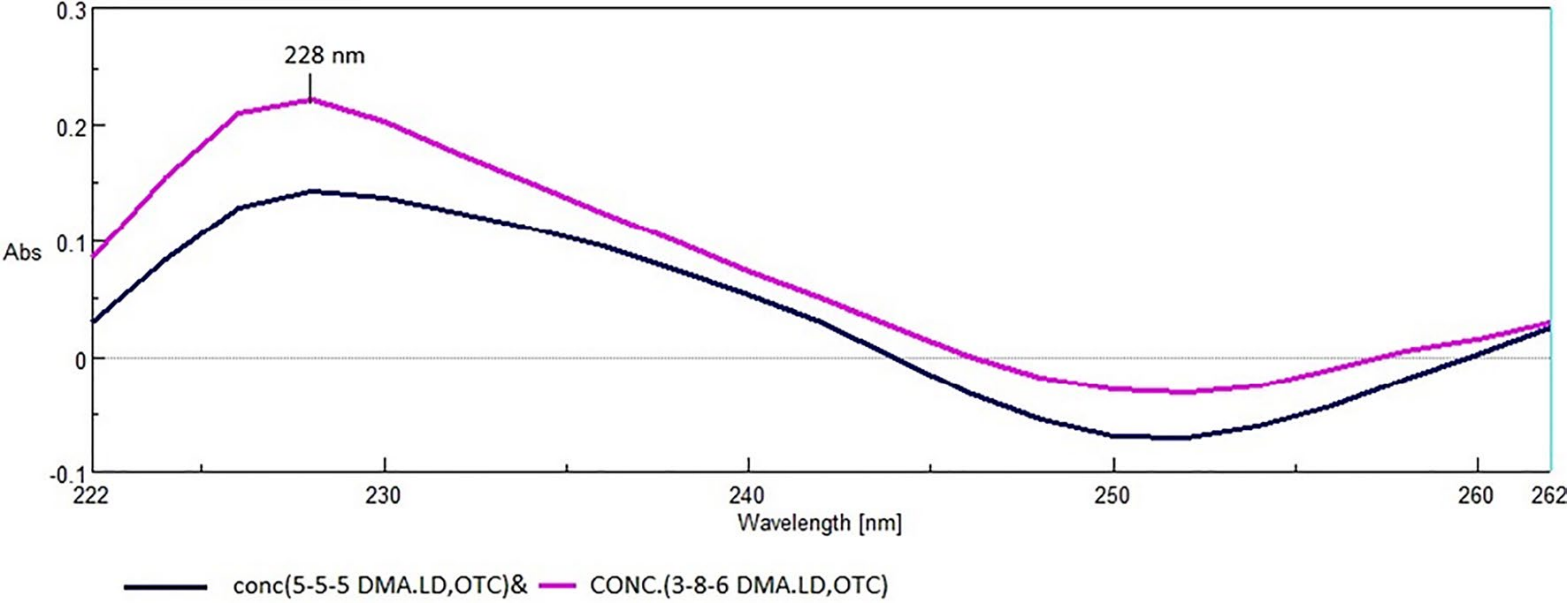
First order spectra of laboratory prepared mixtures of Oxytetracycline, Lidocaine and 2,6- dimethylaniline after removal of oxytetracycline and after division by normalized spectrum of impurity (derivative ratio method)

This method involves the division of the mixture (after removal of OTC) by normalized divisor of DMA then the first derivative of the obtained spectrum (shown in Fig. 5) was computed. The amplitudes of the resulting spectra were measured at 228 nm and related to the concentration.

### Validation

ICH guidelines were adhered to during the validation process of the proposed methods. The validation process included evaluating limit of quantification (LOQ), limit of detection (LOD), accuracy, precision, linearity and range, and selectivity. A comprehensive overview of the validation parameters can be found in Table 2.

**Table 2:** The assay validation results for the suggested spectrophotometric methods for determining OTC, LD, and DMA and analysis of dosage form

Drug	OTC			LD		DMA		
Resolution technique	− 360 nm	CV	Conc. V	Ratio difference (218 nm-230 nm)	Derivative ratio (228 nm)	CM 237 nm	CV	Conc. V
Range µg/mL			4–34	1.5–9	1–9	1.3–12		
Linearity								
Slope	0.0295	1.0033	–	0.409	0.0265	0.0848	0.9872	–
Intercept	0.0009	0.0805	–	0.1684	0.0107	0.0032	0.0514	–
Correlation coefficient (r)	0.9999	1	–	0.9999	0.9999	0.9999	0.9999	–
Accuracy^a^ (mean ± SD)	99.88 ± 0.833	98.64 ± 0.50	100.44 ± 0.410	99.37 ± 0.83	100.36 ± 0.94	99.8 ± 0.97	100.42 ± 0.66	100.48 ± 0.51
Selectivity^b^	99.84 ± 0.97	100.4 ± 0.92	99.84 ± 0.84	100.05 ± 1.5	99.4 ± 1.06	100.7 ± 1.04	100.33 ± 1.23	100.12 ± 1.32
Precision								
Repeatability RSD%^c^	0.770	0.476	0.374	0.553	0.375	0.25	0.422	0.229
Intermediate precision RSD% ^d^	1.150	1.070	0.410	0.928	0.510	0.990	1.630	0.301
LOQ (µg/mL)	1.35	0.837	–	0.243	0.377	0.494	0.506	–
LOD (µg/mL)	0.45	0.230	–	0.080	0.125	0.163	0.167	–
Recovery of pharmaceutical preparations	100.033 ± 0.26	99.54 ± 0.69	99.83 ± 0.84	100.36 ± 1.2	98.8 ± 1.07	–	–	–

^a^ Six concentrations: Each concentration of every analyte was repeated three times

^b^ The mean recovery ± standard deviation were calculated from three replicates of five laboratory-prepared mixtures of OTC, LD, and DMA in each set

^c^ Repeatability (n = 3), The average of three concentrations of analytes (5,17,20 µg/mL OTC, 3.5,5,7 µg/mL LD and 5,7,10 µg/mL DMA) was measured three times on same day

^d^ Intermediate precision (n = 3), An average of three concentrations of analytes (5,17,25 µg/mL OTC, 3.5,4,7 µg /mL LD and 1.5,7,10 µg/mL was measured three times on three different days

e Spectropan 5 commercial veterinary dosage form manufactured by Pharma Swede and labeled to contain 1 mg LD and 54 mg OTC

### Chemometric methods

**Fig. 6:**
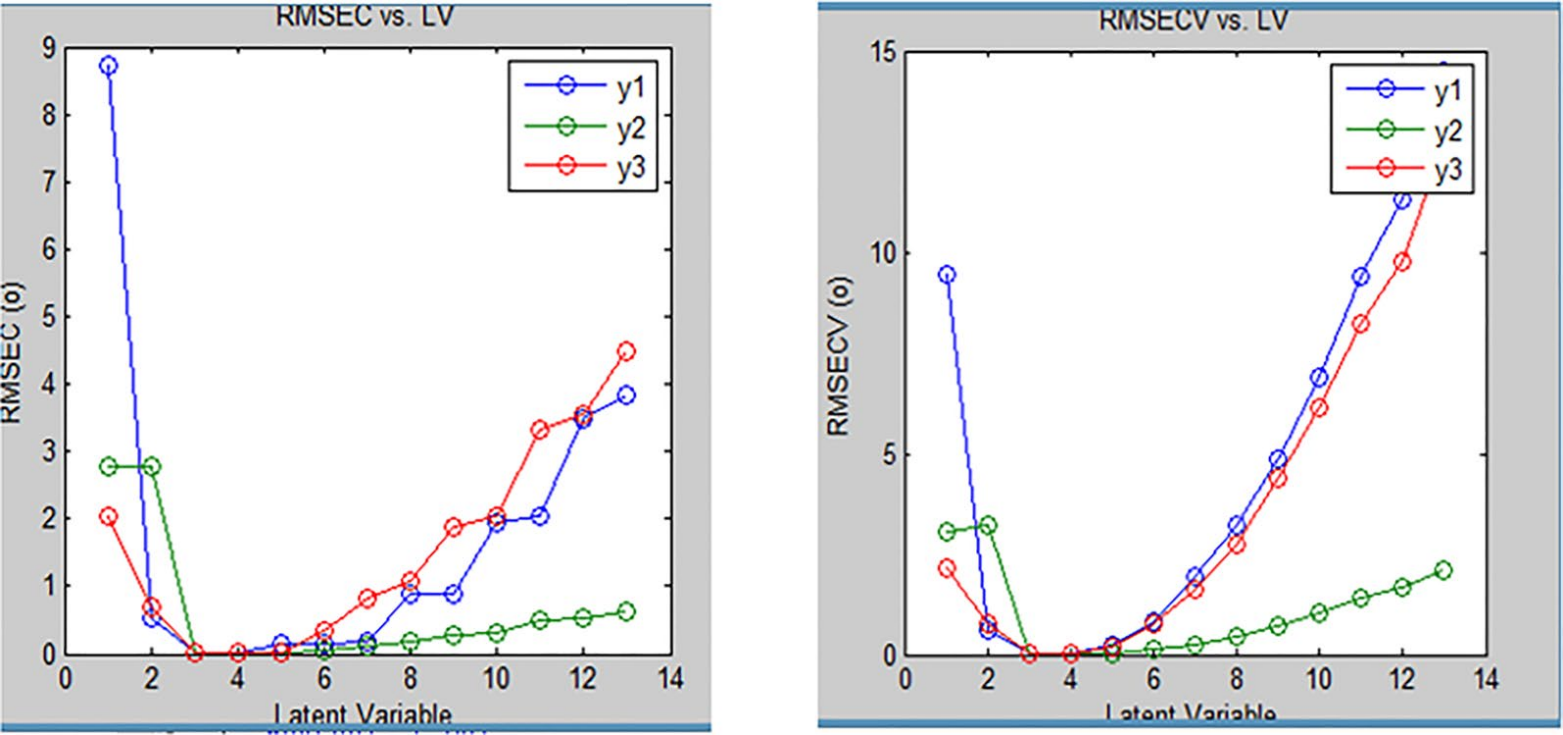
RMSECV and RMSEC plot results of the calibration set as a function of latent variables (LVs) number utilized to construct the PLS model for determination of Oxytetracycline (blue), Lidocaine (green) and 2,6- dimethylaniline (Red)

**Fig. 7:**
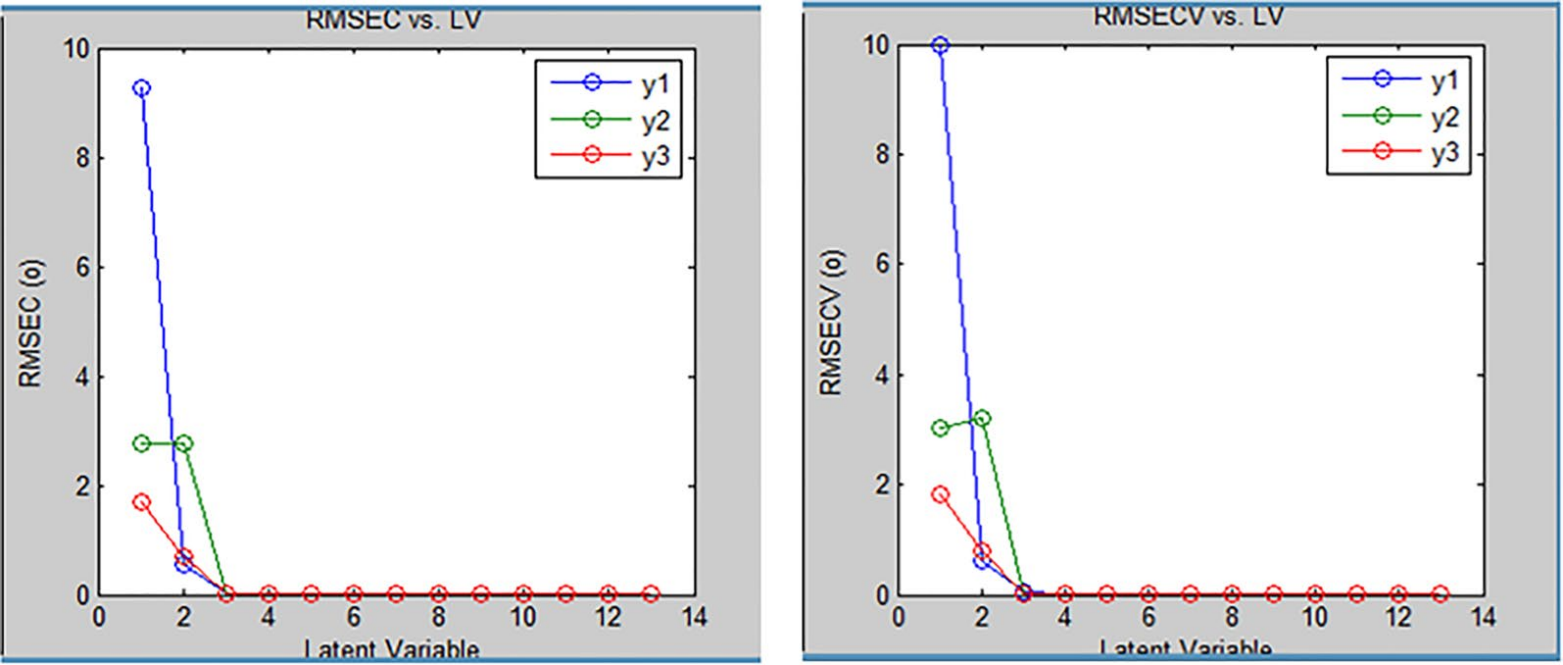
RMSECV and RMSEC plot of results of the calibration set as a function of latent variables (LVs) number utilized to construct the PCR model for Oxytetracycline(blue), Lidocaine(green) and 2,6- dimethylaniline

**Fig. 8:**
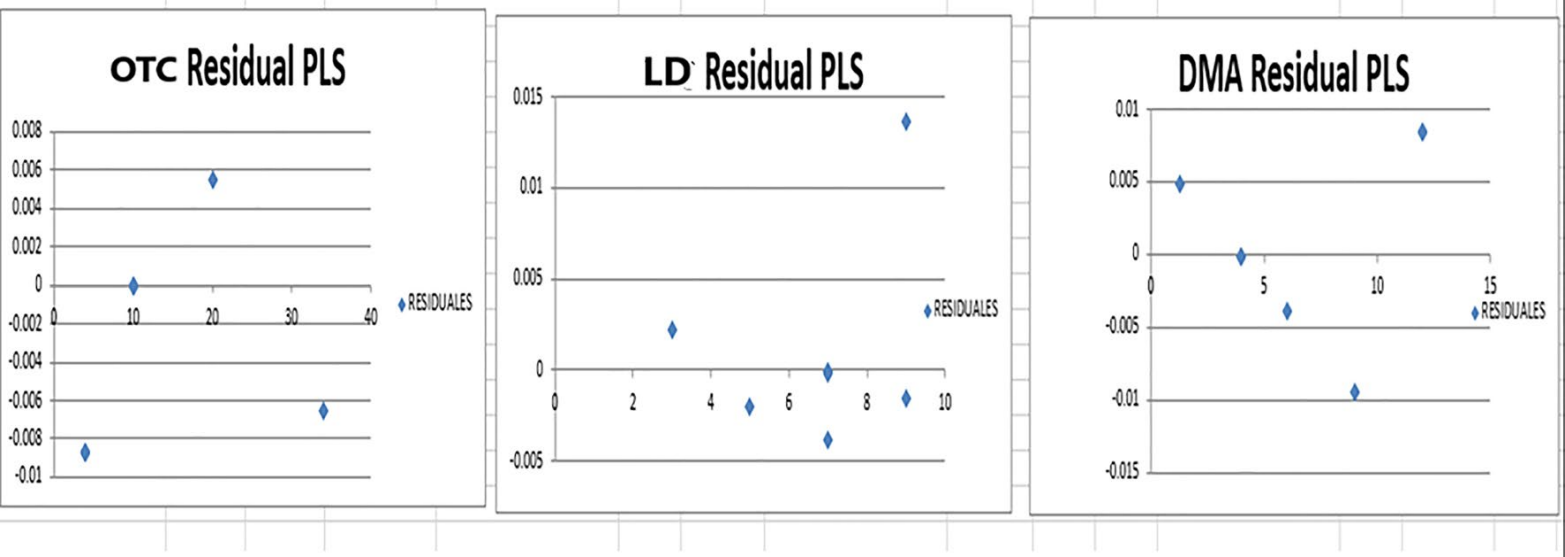
PLS Residual concentrations of OTC, LD and DMA

**Fig. 9:**
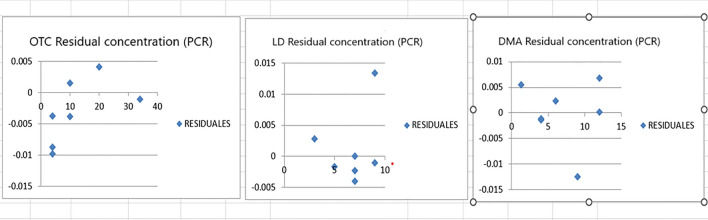
PCR Residual concentrations of OTC, LD and DMA

In our suggested method numerous experimental trials were conducted to achieve the most suitable predictive model and attain optimal results. Prior to constructing the model, determining the ideal number of components was crucial. Preserving an excessive number of factors would lead to an accumulation of noise in the data. Conversely, a low number of preserved factors could lead to the omission of crucial data used for calibration. Therefore, it was important to determine a suitable number of factors that best fit the experimental data without resulting in overfitting [30]. A total of seven mixtures including OTC, DMA, and LD were made by diluting various amounts of the corresponding working solutions into a 10-mL volumetric flask to validate this method. The remaining volume was completed with acetonitrile. The generated mixtures were then subjected to the suggested models in order to predict the concentrations of the drugs under study. The study employed a cross-validation technique where one sample was eliminated at a time. For evaluation of the accuracy and precision of the predictions, the % recovery of each concentration, RMSEC and the RMSECV were calculated. The actual concentrations in the calibration samples were compared to the expected concentrations of each component in each sample. The recoveries, RMSEC and RMSECV provided insights into the reliability and correctness of the predictions. These evaluations were performed iteratively, recalculating the RMSEC and RMSECV recoveries while incorporating each new factor into the PLS and PCR models as shown in Figs. 6 and 7. The developed PLS and PCR models statistical parameters are found in Tables 3 and 4. PLS and PCR residual concentrations of OTC, LD and DMA are shown in Figs. 8 and 9.

**Table 3:** Statistical parameters for determining OTC, LD, and DMA simultaneously with optimized PLS and PCR methods

Parameters of interest	PCR			PLS		
	OTC	LD	DMA	OTC	LD	DMA
Conc. range µg/mL	4.0–34.0	1.0–9.0	1.3–12.0	4.0–34.0	1.0–9.0	1.3–12.0
No. of factors	13					
Predicted conc. versus actual conc. Plot						
Intercept^*^	0.0058	0.0042	0.0003	0.0016	0.0036	0.0003
Slope^*^	1.0002	1.0008	1.0004	1.0003	1.0007	1.0002
Correlation coefficient (r)^a^	1	1	1	1	1	1

^*^ Data showing a straight line plotting each component expected concentration against the validation set actual concentrations

**Table 4:** % recoveries of OTC, LD and DMA by the proposed PCR and PLS Methods in validation set

OTC			LD			DMA		
R%^a^			R%^a^			R%^a^		
True conc., μg/mL	PCR	PLS	True conc., μg/mL	PCR	PLS	True conc., μg/mL	PCR	PLS
4.0	99.7	99.0	9.0	100.2	100.2	4.0	99.96	99.99
34.0	99.9	99.9	3.0	100.0	100.0	12.0	100.2	100.1
10.0	99.0	100.0	9.0	99.9	99.9	6.0	100.03	99.93
10.0	100.0	100.1	7.0	100.0	99.9	12.0	100.5	100.0
4.0	99.7	99.8	7.0	99.0	99.0	1.3	101.0	101.0
4.0	99.9	99.7	5.0	99.96	99.9	9.0	99.8	99.8
20.0	100.0	100.0	7.0	99.7	99.9	4.0	100.0	99.9
Mean ± S.D	99.71 ± 0.35	99.83 ± 0.40		99.82 ± 0.41	99.86 ± 0.41		100.21 ± 0.45	100.1 ± 0.42

a Average of three experiments

### Statistical comparisons

A statistical comparison was conducted between the reported methods [14, 31] and the proposed univariate and multivariate spectrophotometric methods for determining OTC, DMA, and LD as shown in Table 5.

**Table 5:** A statistical comparison between the reported approach and the suggested methods to determine DMA, LD, and OTC in bulk powder

Resolution technique	Drug	method	mean	SD	N	Variance	Student's *t* test^*a*^ (2.23) *, (2.26) **	F test^*a*^ (5.05) *, (5.19) **
–	OTC	360 nm	99.95	0.76	6	0.58	0.16	2.7
		CV	100.09	1.14	6	1.3	0.32	1.6
		Conc. V	99.97	1.16	6	1.34	0.12	1.6
chemometric methods		PCR	99.99	0.5	7	0.16	0.23	3.24
		PLS	99.91	0.5	6	0.16	0.025	3.2
–	LD	Ratio difference	100.2	0.93	6	0.86	0.56	1.4
		Derivative ratio	100.65	0.52	6	0.27	1.9	2.4
Chemometric methods		PCR	99.96	0.5	7	0.25	0.11	2.6
		PLS	100.01	0.50	7	0.25	0.24	2.6
CM	DMA	237 nm	100.05	0.68	6	0.46	0.15	2.6
–		CV	99.7	1.05	6	1.10	0.53	1.01
–		Conc. V	99.7	0.76	6	0.58	0.78	2.1
Chemometric methods		PCR	100.2	0.5	7	0.25	0.14	4.8
		PLS	100.10	0.50	7	0.25	0.06	4.8
Reported method		OTC^a^	99.9	0.9	6	0.81		
		LD^a^	99.92	0.8	6	0.64		
		DMA^b^	100.13	1.1	6	1.21		

For spectrophotometric* and chemometric** approaches, the values in parenthesis represent the equivalent theoretical values of t and F at p = 0.05

^a^ Spectrophotometric method [14]

^b^ Spectrophotometric method [31]

One-way ANOVA was used to determine if there were any significant differences between suggested methods and the reported one [32]. This includes the following steps:

- Define the Groups: The groups consisted of the proposed spectrophotometric methods and the reported standard method. These groups were compared to assess whether the suggested methods perform similarly to the established one.
- Data Collection: Absorbance measurements of the drug were taken for each method, with multiple replicates for each group to ensure the reliability and consistency of the data.
- Perform ANOVA: A one-way ANOVA was used to compare the mean absorbance values across the different spectrophotometric methods. This statistical test evaluates whether the observed differences in absorbance are due to the methods themselves or just random variation within the data.
- Interpret Results: If the p-value from the ANOVA test was less than 0.05, it would indicate a significant difference in the absorbance measurements between the proposed methods and the reported standard, suggesting that the methods are not equivalent. If the p-value was greater than 0.05, it would suggest that there were no significant differences between the methods, indicating that the proposed methods are comparable to the reported one.

As shown in Table 6 Statistical comparison using One-way ANOVA were performed between the suggested univariate spectrophotometric methods and the reported methods [14, 31] which indicates no statistically significant difference between the proposed and reported methods. Additionally, as shown in Table 7, this approach ensures that the proposed spectrophotometric methods are evaluated for their accuracy and reliability against an established method, confirming their suitability for use in similar analytical applications the pharmaceutical dosage form was subjected to one-way ANOVA to compare the suggested method with the reported HPLC method [15].

**Table 6:** ANOVA (single factor) results to compare the proposed methods for the determination of OTC, LD and DMA in pure powder form and the reported method

Source of variation		SS a	df b	MS	F cal. C	P-value	F cr. d
^OTC^	Between Groups	1.3	3	0.42	0.44	0.72	3.1
	Within Groups	19.1	20	0.95			
	Total	20.4	23				
^LD^	Between Groups	0.012	2	0.006	0.006	0.99	3.7
	Within Groups	13.5	15	0.89			
	Total	13.5	17				
^DMA^	Between Groups	0.48	3	0.16	0.19	0.90	3.1
	Within Groups	16.8	20	0.84			
	Total	17.3	23				

a Sum of squares

b Degree of freedom within and between groups

c Mean square

d Calculated F

**Table 7:** ANOVA (single factor) results to compare the proposed methods and the reported method for the determination of OTC and LD in pharmaceutical dosage form

Source of Variation		SS^a^	df^b^	MS^c^	F cal.^d^	P-value	F cr.^e^
OTC	Between Groups	0.69	3	0.23	0.71	0.57	4.06
	Within Groups	2.60	8	0.33			
	Total	3.29	11				
LD	Between Groups	3.38	2	1.69	1.27	0.35	5.14
	Within Groups	7.94	6	1.32			
	Total	11.33	8				

^a^ Sum of squares

^b^ Degree of freedom between and within groups

^c^ Mean square

^d^ Calculated F

^e^ Critical (tabulated) value for F at p = 0.05

## Conclusion

The lack of a distinct peak in LD zero-order spectrum made it difficult to determine the drug directly using spectrophotometry with adequate accuracy and precision, fortunately, our method was successful in separating LD and each component of the ternary mixture by accurate and precise spectrophotometric methods without the interference of two other components and without dependence on LD shoulder peak. The results obtained from all suggested spectrophotometric methods used demonstrate that no significant difference between the proposed methods, which measure OTC, LD, and DMA, and the reported methods. To further validate the efficacy of the developed method, additional univariate and multivariate spectrophotometric techniques were developed and evaluated to determin**e** the combination of OTC, LD, and DMA in laboratory mixtures. Also, LD and OTC were determined in marketed dosage form and proved to be precise, accurate and selective. Importantly, by comparing all of the developed methods to each other and to the reported methods, no significant difference was found. This indicates their reliability and interchangeability in practical applications. As a result, these methods can be readily employed in laboratories of quality control to facilitate rapid determination of the mentioned drugs.

## Data Availability

All data generated or analyzed during this study are included in this published article.

## Abbreviations

LD: Lidocaine HCl
OTC: Oxytetracycline HCl
DMA: 2,6- Dimethylaniline
SPME: Solid phase microextraction
GC/MS: Gas chromatography/mass spectrometry
HPLC: High performance liquid chromatography
TLC: Thin layer chromatography.
CV: Constant value
CNV: Concentration value
ICH: International council for harmonization
RSD: Relative standard deviations
LOQ: Limit of quantitation
LOD: Limit of detection
RMSEC: Root mean square error of calibration
RMSECV: Root mean standard error of validation
PLS: Partial least square
PCR: Principle component regression
CM: Constant multiplication
IARC: International agency for research on cancer
ANOVA: Analysis of variance
